# Two Decades of Progress in the Prevention and Treatment of Colorectal Cancer: From Aspirin to Targeted Therapy

**DOI:** 10.3390/biomedicines14071472

**Published:** 2026-06-29

**Authors:** Lovyanne Vergel De Dios, Salique Hassan Shaham, Puneet Vij, Manish K. Tripathi

**Affiliations:** 1 School of Medicine, University of Texas Rio Grande Valley, Edinburg, TX 78541, USA; lovyanne.vergeldedios01@utrgv.edu; 2 Department of Medicine and Oncology, School of Medicine, University of Texas Rio Grande Valley, McAllen, TX 78504, USA; salique.shaham@utrgv.edu; 3 South Texas Center of Excellence in Cancer Research, School of Medicine, University of Texas Rio Grande Valley, McAllen, TX 78504, USA; 4 Department of Pharmaceutical Sciences, St. John’s University, Queens, NY 11439, USA; puneet.vij08@outlook.com

Colorectal cancer (CRC) remains the third most commonly diagnosed malignancy and the second leading cause of cancer-related mortality worldwide [1]. Over the past two decades, substantial advances in screening, prevention, and systemic therapies have reshaped the clinical management of CRC, leading to improved outcomes in certain populations. Although global trends demonstrate a decline in CRC incidence over the past decade, there has been a notable increase in cases among individuals under 50 years of age, with approximately 10% of new CRC diagnoses occurring in this population [2]. Traditionally, risk assessment has relied primarily on family history and age. However, as nearly 75% of patients with early-onset CRC lack a family history of the disease, the necessity for enhanced strategies in early detection and treatment has become increasingly critical [2].

CRC is a multifactorial disease arising from a complex interplay of genetic predispositions, environmental influences, and lifestyle choices. The pathogenesis of CRC can progress through at least three distinct molecular pathways: chromosomal instability (CIN), microsatellite instability (MSI), and CpG island methylator phenotype pathways [3]. The CIN pathway, present in 65% to 70% of sporadic CRCs, involves a stepwise accumulation of mutations in APC, KRAS, and TP53 [3]. These genetic alterations give rise to three principal tumor subtypes: adenocarcinoma, mucinous adenocarcinoma, and signet ring cell carcinoma. Tumor heterogeneity, subdivided into genetic and non-genetic, adds another layer of complexity to cancer treatment. CRC can also arise from COX-2 overexpression, supporting the use of nonsteroidal anti-inflammatory drugs (NSAIDs) such as aspirin, which has been widely investigated for its chemopreventive role through inhibition of prostaglandin-mediated inflammation and tumorigenesis in CRC prevention. Additional key biomarkers, such as BRAF, NRAS, and PIK3CA, further refine tumor specificity and therapeutic implications [4]. These advances have fundamentally altered how CRC is conceptualized and managed. However, they have also exposed important limitations in current approaches. Rising rates of early-onset CRC, persistent disparities in outcomes, and the development of therapeutic resistance suggest that future progress will depend not simply on identifying new therapies but on integrating precision prevention and molecularly guided treatment strategies into routine clinical practice. In our view, this transition toward precision oncology represents the defining challenge of the next decade of CRC research.

Despite meaningful advances in our understanding of CRC biology, survival outcomes remain far from optimal. Five-year survival rates have improved in high-income countries, approaching ~60% overall, but remain significantly lower for patients with advanced or metastatic disease [5]. In contrast, differences across racial and ethnic groups are greater. Although Asian and Hispanic Americans experience the lowest incidence rates (28.6 and 32.5 annual cases per 100,000 people, respectively), survival gaps remain concerning [2]. Even more striking is the disproportionate burden among Alaska Native, American Indian, and Black populations, with incidence rates of 88.5, 46.0, and 41.7 per 100,000, respectively. Black patients also face a significantly lower 5-year survival rate (54%) compared with White patients (61%) [2]. These disparities highlight that advances in CRC treatment have not been experienced equally across patient populations. Precision oncology must therefore extend beyond biomarker discovery to ensure equitable implementation of molecular diagnostics, access to treatment opportunities, and timely delivery of emerging therapies.

The need for new therapies is most pronounced in metastatic CRC (mCRC), where traditional management strategies, such as surgery and chemotherapy, are often not viable. Combination therapies enable simultaneous multipathway targeting, leading to a more robust treatment strategy. The Food and Drug Administration (FDA) has approved multiple immunotherapy drugs for the initial treatment of mCRC: nivolumab (Opdivo) and ipilimumab (Yervoy) [6]. Nivolumab, a monoclonal antibody against the programmed cell death immune checkpoint inhibitor (PD-1 receptor), is used in combination with ipilimumab, a monoclonal antibody against the immune checkpoint inhibitor on T-cells (CTLA-4) [6]. Although combination therapy has been clinically proven effective in slowing the progression of mCRC tumors, its effectiveness is limited to those with deficient mismatch repair (dMMR) or high-risk microsatellite instability (MSI-H) tumors [6]. The success of checkpoint inhibition in MSI-H/dMMR disease illustrates both the promise and limitations of precision oncology: remarkable outcomes are achievable when effective biomarkers exist, yet the majority of CRC patients remain outside the reach of these therapeutic advancements. Expanding these benefits to biomarker-negative populations remains a major unmet need.

Recent studies have highlighted promising new genetic targets for CRC treatment. The melanoma cell adhesion molecule (MCAM) becomes soluble and tumorigenic when released from the cell membrane by ADAM17, whose levels are regulated by NOX1 [7]. Consequently, NOX1 acts as an indirect regulator of soluble MCAM. In a recent study, treatment of CRC tumor-bearing mice with NOX1 inhibitors reduced MCAM production but also abolished the anti-tumor effects of anti-MCAM antibodies. In contrast, anti-ADAM17 inhibitors reduced both angiogenesis and tumor growth in vivo without negating pre-existing anti-tumor mechanisms [7]. These findings are particularly relevant because CMS4 CRC continues to lack effective targeted therapies. Rather than representing another isolated molecular discovery, the NOX1/ADAM17 axis exemplifies the next generation of therapeutic targets aimed at overcoming the limitations of the current treatment model.

The Hippo signaling pathway has been increasingly implicated in cancer tumorigenesis and metastatic progression. Deregulation of its core components, including LATS1, LATS2, MST1, YAP, and TAZ, has been associated with the development of secondary malignancies [8]. Although mutations within these core elements are relatively uncommon, the pathway’s extensive interplay with other major signaling networks underscores its potential to profoundly influence oncogenic processes when dysregulated [8]. Although clinical applications remain investigational, targeting Hippo-associated signaling networks may provide future opportunities to limit metastatic spread and overcome treatment resistance.

The future of CRC precision medicine may extend beyond tumor genomics alone. Emerging evidence suggests that host-associated factors, particularly the gut microbiome, influence therapeutic efficacy and the development of treatment resistance. Studies in pancreatic ductal adenocarcinoma (PDAC) have shown that microbiome composition contributes to chemoresistance through drug metabolism and immune modulation [9]. While these observations arise from another gastrointestinal malignancy, they highlight an important concept for CRC, namely that treatment response is determined not only by tumor biology but also by the surrounding microbial environment. Incorporating microbiome profiling and microbiome-targeted interventions into precision oncology frameworks may offer new opportunities to improve treatment outcomes and overcome resistance in CRC.

As precision medicine evolves, population-specific molecular profiling will become increasingly important. An epidemiological analysis of CRC in Serbia, based on national cancer registry data, revealed that among 6369 patients tested, over half harbored mutations in either KRAS or NRAS, while only 9.54% exhibited the BRAF V600 mutation [4]. These findings emphasize that therapeutic strategies developed in one population may not be universally applicable. Expanding global molecular surveillance efforts will be essential to ensure equitable implementation of targeted therapies and avoid widening existing disparities in CRC outcomes.

Standardized chemotherapeutic regimens for CRC, including irinotecan, oxaliplatin, and 5-fluorouracil, have led to meaningful improvements in patient survival. However, these treatments are limited by systemic toxicities and the emergence of drug resistance [10]. In response, targeted therapies directed against pathways such as the epidermal growth factor receptor (EGFR), vascular endothelial growth factor (VEGF), and Hedgehog have emerged as promising alternatives, offering greater precision and less harm to healthy tissues [10]. Additionally, novel targets, including transforming growth factor-beta (TGF-β), insulin-like growth factor/insulin-like growth factor 1 receptor (IGF/IGF1R), Notch, and the Wnt/β-catenin pathway, represent important areas for continued research and therapeutic development [10].

In summary, colorectal cancer (CRC) remains a complex and pressing public health challenge, marked by rising incidence in younger populations and persistent outcome disparities across demographic groups. Advances in molecular characterization and therapeutic development have transformed CRC management, shifting the field from broadly applied preventive and cytotoxic strategies toward increasingly individualized approaches guided by molecular biomarkers. However, significant challenges remain. Most patients with metastatic CRC remain ineligible for currently approved immunotherapies, while acquired resistance frequently limits the durability of targeted treatments. Furthermore, the biological mechanisms underlying early-onset CRC remain incompletely understood, and disparities in screening uptake, molecular testing, and access to specialized care continue to impede equitable progress. Addressing these barriers will require multidisciplinary collaboration that bridges epidemiology, translational science, and clinical investigation. Success in the next phase of CRC research will depend not only on identifying new therapeutic targets but also on integrating prevention, early detection, molecular diagnostics, and innovative therapeutics into a cohesive precision oncology framework. Only then can the substantial gains achieved over the past two decades translate into meaningful reductions in the global burden of colorectal cancer.
